# Examining allostatic load, neighborhood socioeconomic status, symptom burden and mortality in multiple myeloma patients

**DOI:** 10.1038/s41408-022-00648-y

**Published:** 2022-04-01

**Authors:** Samilia Obeng-Gyasi, Noah Graham, Shaji Kumar, Ju-Whei Lee, Susanna Jacobus, Matthias Weiss, David Cella, Fengmin Zhao, Edward H. Ip, Nathaniel O’Connell, Fangxin Hong, Devin J. Peipert, IIana. F. Gareen, Lava R. Timsina, Robert Gray, Lynne I. Wagner, Ruth C. Carlos

**Affiliations:** 1 The Ohio State University, Columbus, OH USA; 2 Dana-Farber Cancer Institute, ECOG-ACRIN Biostatistics Center, Boston, MA USA; 3 Mayo Clinic, Rochester, MN USA; 4 ThedaCare Cancer Care, Appleton, WI USA; 5 Robert H. Lurie Comprehensive Cancer Center, Northwestern University, Chicago, IL USA; 6 Wake Forest University School of Medicine, Winston-Salem, NC USA; 7 Northwestern University Feinberg School of Medicine, Chicago, IL USA; 8 Brown University Department of Epidemiology and Center for Statistical Sciences, Providence, RI USA; 9 Indiana University School of Medicine, Indianapolis, IN USA; 10 University of Michigan Comprehensive Cancer Center, Ann Arbor, MI USA

**Keywords:** Health services, Prognosis

## Abstract

The objective of this study is to examine the association between neighborhood socioeconomic status (nSES) and baseline allostatic load (AL) and clinical trial endpoints in patients enrolled in the E1A11 therapeutic trial in multiple myeloma (MM). Study endpoints were symptom burden (pain, fatigue, and bother) at baseline and 5.5 months, non-completion of induction therapy, overall survival (OS) and progression-free survival (PFS). Multivariable logistic and Cox regression examined associations between nSES, AL and patient outcomes. A 1-unit increase in baseline AL was associated with greater odds of high fatigue at baseline (adjusted OR [95% CI] = 1.21 [1.08–1.36]) and a worse OS (adjusted hazard ratio, [95% CI] = 1.21 [1.06–1.37]). High nSES was associated with worse baseline bother (middle OR = 4.22 [1.11–16.09] and high 4.49 [1.16–17.43]) compared to low nSES. There was no association between AL or nSES and symptom burden at 5.5 months, non-completion of induction therapy or PFS. Additionally, there was no association between nSES and OS. AL may have utility as a predictive marker for OS among patients with MM and may allow individualization of treatment. Future studies should standardize and validate AL patients with MM.

## Introduction

The American Cancer Society estimates 34,920 people will be diagnosed with multiple myeloma (MM) in 2021 and 12,410 will succumb to the disease [1]. Despite significant improvements in the diagnosis and treatment of MM, social determinants of health continue to influence clinical outcomes in this population [2]. Social determinants of health (SDH) describe environmental, psychosocial, biological, and behavioral characteristics that influence overall health and clinical outcomes (e.g., diagnosis, treatment, and survival) [3]. For instance, patients with MM living in counties with high poverty rates have worse mortality rates than those in areas with lower poverty rates [4]. Probable explanations for the influence of SDH in patients with MM rests on a complex interplay between resource availability (e.g., access to healthcare), environmental exposures, adverse living conditions, genetics, and psychosocial factors [5, 6]. Emerging frameworks suggest life experiences influenced by socially patterned exposures such as SDH (e.g., socioeconomic position or social isolation) may exert their effects on health through stress-related pathways such as the hypothalamic-pituitary-adrenal axis (HPA) and the sympathetic-adrenal-medullary system (SAM) [7, 8].

Allostatic load (AL) is a composite score that measures the cumulative effects of chronic stress on physiology [9]. Specifically, AL posits that prolonged exposure to stressful interpersonal and environmental circumstances leads to multisystem physiologic dysregulation resulting in increased morbidity and mortality [7]. Biomarkers used to calculate AL are reflective of HPA (e.g., cortisol), the SAM (e.g., norepinephrine) and their downstream effects on the immune, metabolic and cardiovascular systems [10, 11]. Elevated AL has been linked to SDH such as low socioeconomic status and educational attainment [12–15]. Moreover, increased AL has been associated increased disease-specific and overall mortality in cancer patients [16, 17]. Notably, individual components of AL have been independently associated with tumorigenesis, mortality, and patient-reported adverse events [18–20].

Although there have been some studies evaluating the association between SDH and clinical outcomes among patients with MM, there have been no studies examining the relationship between AL and clinical outcomes [2, 4, 21–23]. The objective of this study is to understand the relationship between neighborhood socioeconomic status (nSES) or baseline AL and symptom burden (i.e., pain, fatigue, and bother), induction therapy completion and overall survival among patients with MM enrolled in an ECOG ACRIN clinical trial.

## Methods

### Data source

ECOG–ACRIN E1A11 (NCT01863550) was a multicenter, open-label, phase 3 randomized controlled trial comparing bortezomib, lenalidomide and dexamethasone (VRd) to carfilzomib, lenalidomide, and dexamethasone (KRd) in newly diagnosed symptomatic standard-risk patients with MM. Induction therapy for both study arms lasted for 36 weeks. For the VRd arm, induction therapy included 12 cycles of 3 weeks, where patients received 1·3 mg/m² of bortezomib subcutaneously or intravenously on days 1, 4, 8, and 11 of cycles 1–8, and day 1 and day 8 of cycles nine to twelve, 25 mg of oral lenalidomide on days 1–14, and 20 mg of oral dexamethasone on days 1, 2, 4, 5, 8, 9, 11, and 12. In the KRd arm, for nine cycles of 4 weeks, patients received 36 mg/m² of intravenous carfilzomib on days 1, 2, 8, 9, 15, and 16, 25 mg of oral lenalidomide on days 1–21, and 40 mg of oral dexamethasone on days 1, 8, 15, and 22. For post-induction treatment, study subjects were randomized to lenalidomide for 2 years maintenance or indefinitely until progression or excessive toxicity. In the E1A11 trial, the study endpoints were progression-free survival (PFS) and overall survival (OS) [24].

### Neighborhood socioeconomic status (nSES)

The neighborhood level SES index was generated by linking the patient’s home zip code at registration to census tract data using 2019 American Community Survey (ACS) from U.S. Census database [25, 26]. The nSES index was created by the Agency for Health Research and Quality (AHRQ) and its components include employment, income, poverty, wealth, education, and crowding at the census tract level [27–29]. For each component linking with a zip code representing multiple census tracts, the data was aggregated as a mean to represent an estimate for that zip code [30]. Lower nSES index values represent higher levels of neighborhood deprivation.

### Allostatic load

The biomarkers for allostatic load were selected based on their availability in the E1A11 data set and their frequency of use in prior studies evaluating AL [31]. AL biomarkers focused on the metabolic, renal, and immune physiologic systems. A total of seven biomarkers were used to calculate AL. Biomarkers from the metabolic system included body mass index (BMI) (kg/m^2^), albumin, and alkaline phosphatase. The renal system biomarkers were creatinine and creatinine clearance, and immune system biomarkers were C-reactive protein (CRP) and white blood cell count (WBC). All AL biomarkers were collected after study enrollment but prior to beginning induction therapy.

The AL score was calculated as a composite of these seven biomarkers. Patients received one point toward the AL score for each biomarker that fell in the “worst” sample quartile for that biomarker. Specifically, values in the lowest sample quartile earned a point for the albumin and creatine clearance components of AL, whereas values in the lowest and highest sample quartiles earned a point for the BMI component; values in the highest sample quartile earned a point for the alkaline phosphatase, creatinine, CRP, and WBC components. The total AL score is the sum of the seven biomarker scores, and ranges from 0 to 7; patients missing values for any of the seven biomarkers did not receive a score. Another version of the AL score was also derived for sensitivity analysis, in which previously established clinical cut points were used to classify values of each biomarker as normal or abnormal; patients received a point toward the AL score for each abnormal biomarker. These cut points used for classification are detailed in Supplementary Table 1.

### Symptom burden

Symptom burden was evaluated using the Functional Assessment of Cancer Therapy-General (FACT-G) survey and the Multiple Myeloma Subscale (MMS). FACT-G items analyzed included GP4 (“I have pain”) and GP5 (“I am bothered by the side-effects of treatment”). One MMS item was analyzed – HI7 (“I feel fatigued”). All three items are measured on a 5-point Likert scale (0-Not at all; 1-A little bit; 2-Somehow; 3-Quite a bit; 4-Very much) and were evaluated at baseline and 5.5 months after induction registration.

### Statistical analysis

All analyses of the current study were post-hoc analyses conducted among E1A11 patients with available (non-missing) AL and nSES scores. Study endpoints were symptom burden assessed at baseline and 5.5 months after study entry, non-completion of induction therapy, progression-free survival (PFS), and overall survival (OS). Scores from each of GP4, GP5, and HI7 were dichotomized into low (0–2) and high (>=3) for analysis. Analyses of induction therapy non-completion excluded all patients that discontinued treatment due to disease progression or death, and non-completion was defined as having gone off treatment for any reason other than completion per protocol. Overall survival was defined as the interval from induction registration to death from any cause, or to last follow up for patients still living. Progression-free survival was defined as the interval from induction registration to first documented disease progression or death, or to last follow up for patients still living without progression.

Multivariable logistic regression was used to estimate the effects of AL and nSES on high pain, high bother, and high fatigue, at baseline and at 5.5 months, adjusting for age, sex, race, disease stage (ISS stage), ECOG performance status (PS), and treatment arm. Models of symptom burden at 5.5 months were adjusted for the same covariates, plus the baseline score. PFS and OS were analyzed via the Kaplan–Meier method, and log-rank tests were used to test for differences in PFS and OS by AL and nSES. Multivariable Cox regression was also used to estimate the effects of AL and nSES on PFS and OS while adjusting for the same covariates previously mentioned, and additionally adjusting for high-bother, -pain, and -fatigue at baseline, and genetic high risk defined as the presence of the t(4;14) and/or -1q genetic abnormalities. Genetic risk was included due to its association with clinical outcomes [32]. Adjustment variables for all models were selected on the basis of data availability and known or hypothesized clinical relevance to the outcome. For all analyses, nSES was analyzed by tertile (“Low”, “Middle”, “High”), with “Low” as the reference category. AL was analyzed as a continuous variable (range [0–7]) for all analyses except log-rank tests, for which it was split into five categories: scores of 0, 1, 2, 3, or >=4. Assumptions for logistic and Cox regression models were verified, and interaction between AL and nSES was explored in all models. Significance level for two-sided testing was set at alpha<0.05.

To assess an alternative method of operationalizing AL, the same primary analyses were conducted using the AL score that was calculated based on clinical cut points for abnormality (as described above) (Supplementary Table 1). Finally, an exploratory analysis to examine bivariate associations between the study endpoints and each AL biomarker individually was conducted (Supplementary Table 2). A forward variable selection procedure was used to assess the predictive value of the composite AL score compared to its individual constituents in modeling OS and PFS.

## Results

Of the 1087 patients in E1A11, 154 (14.2%) were missing an AL or nSES score (*n* = 62 and *n* = 96, respectively), and were subsequently excluded. A total of 933 patients comprised the study cohort. The median age was 65 years (interquartile range [IQR, 58–71]), and most of the study population was white (85.3%), had an ECOG performance status ≤1 (89.7%), and was privately insured or had a combination of private and Medicare insurance (66.9%). The median nSES score was 53.7 (IQR [51.5–56.8]), and the median AL score was 2 (IQR (1–3]) (Table 1).

**Table 1:** Study sociodemographic and clinical characteristics.

	All Patients (*N* = 933)	Arm A (*N* = 466)	Arm B (*N* = 467)
*Age*			
Mean ± SD	64.1 ± 9.3	63.9 ± 9.6	64.3 ± 9.1
Median (Q1, Q3)	65.0 (58.0, 71.0)	64.0 (57.0, 71.0)	65.0 (59.0, 71.0)
*Sex*			
Male	546 (58.5)	271 (58.2)	275 (58.9)
Female	387 (41.5)	195 (41.8)	192 (41.1)
*Race*			
White	769 (85.3)	378 (83.8)	391 (86.9)
Black	113 (12.5)	62 (13.7)	51 (11.3)
Other	19 (2.1)	11 (2.4)	8 (1.8)
Missing/unknown	32	15	17
*Ethnicity*			
Hispanic/Latino	54 (5.9)	28 (6.2)	26 (5.7)
Non-Hispanic	858 (94.1)	427 (93.8)	431 (94.3)
Missing/Unknown	21	11	10
*Disease stage (ISS Stage)*			
I	263 (31.6)	127 (31.0)	136 (32.1)
II	356 (42.8)	175 (43.0)	181 (42.7)
III	213 (25.6)	106 (26.0)	107 (25.2)
Missing/unknown	101	58	43
*Genetic high risk*^*a*^			
Yes	297 (33.6)	147 (33.4)	150 (33.9)
No	586 (66.4)	293 (66.6)	293 (66.1)
Missing/unknown	50	26	24
*ECOG performance status*			
0	379 (40.6)	178 (38.2)	201 (43.0)
1	458 (49.1)	237 (50.9)	221 (47.3)
2	82 (8.8)	42 (9.0)	40 (8.6)
3	14 (1.5)	9 (1.9)	5 (1.1)
*Insurance*			
Private/medicare & private	624 (66.9)	303 (65.0)	321 (68.7)
Medicare/other gov	210 (22.5)	102 (21.9)	108 (23.1)
Medicaid/uninsured	71 (7.6)	45 (9.7)	26 (5.6)
Other/unknown	28 (3.0)	16 (3.4)	12 (2.6)
*Allostatic load score*			
Mean ± SD	3.0 ± 1.0	3.0 ± 1.0	3.0 ± 1.0
Median (Q1, Q3)	2.0 (1.0, 3.0)	2.0 (1.0, 3.0)	2.0 (1.0, 3.0)
*nSES Index*			
Mean ± SD	54.2 ± 4.5	53.9 ± 4.5	54.5 ± 4.4
Median (Q1, Q3)	53.7 (51.5, 56.8)	53.3 (51.3, 56.5)	54.1 (51.6, 57.1)
*Baseline GP4*			
Mean ± SD	1.8 ± 1.4	1.8 ± 1.4	1.8 ± 1.4
N missing	27	14	13
*Baseline GP5*			
Mean ± SD	0.3 ± 0.8	0.4 ± 0.8	0.3 ± 0.8
N missing	74	39	35
*Baseline HI7*			
Mean ± SD	1.6 ± 1.3	1.6 ± 1.3	1.6 ± 1.3
N missing	30	16	14

^a^Genetic high risk is defined as the presence of either t(4;14) or -1q genetic abnormalities.

Table 2a shows rates of high symptom burden at baseline and 5.5 months, and Table 2b shows the adjusted effects of AL and nSES on symptom burden at baseline and 5.5 months. A 1-unit increase in baseline AL was associated with greater odds of high fatigue (odds ratio (OR) [95% CI] = 1.14 [1.01–1.30]) at baseline, when adjusting for other sociodemographic and clinical variables. There was no significant association between AL and high pain or -bother at baseline. However, being in the middle or high tertile of nSES was associated with greater odds of high bother at baseline (4.22 [1.11–16.09] and 4.49 [1.16–17.43], respectively) compared to low nSES. There was no significant association between nSES and high fatigue or high pain at baseline.

**Table 2:** **a** Rates of high symptom burden at baseline and 5.5 months. **b** Summary of effects of AL and nSES on symptom burden at baseline and 5.5 months.

Symptom	Baseline		5.5 Months	
	*N* (%) [95% CI]^a^	*N* missing	*N* (%) *[95% CI]	*N* missing
***(a)*** *Rates of high symptom burden at baseline and 5.5 months*				
High pain	320 (35.3) [32.2–38.5]	27	85 (15.9) [12.9–19.3]	400
High bother	30 (3.5) [2.4–4.9]	74	63 (11.8) [9.2–14.8]	397
High fatigue	227 (25.1) [22.3–28.1]	30	119 (22.1) [18.7–25.9]	395

Endpoint	Baseline		5.5 Months	
	Adjusted odds ratio (95%CI)	*P* value	Adjusted Odds Ratio (95%CI)	P value
*(b) Summary of effects of AL and nSES on symptom burden at baseline and 5.5 months.*				
*High pain*				
AL	1.05 (0.94–1.18)	0.39	0.90 (0.73–1.10)	0.29
nSES^a^	1.13 (0.77–1.64) 0.81 (0.54–1.20)	0.22	1.06 (0.56–2.00) 1.02 (0.51–2.04)	0.98
*High bother*				
AL	1.20 (0.88–1.64)	0.24	0.91 (0.72–1.15)	0.45
nSES	4.22 (1.11–16.09) 4.49 (1.16–17.43)	0.03	0.86 (0.43–1.72) 0.66 (0.31–1.43)	0.57
*High fatigue*				
AL	1.14 (1.01–1.30)	0.04	0.87 (0.72–1.05)	0.15
nSES	1.31 (0.87–1.98) 1.15 (0.75–1.78)	0.43	1.07 (0.60–1.90) 0.96 (0.52–1.76)	0.94

^a^Exact binomial confidence interval.

^b^For nSES, the top line shows effect for middle vs low, and the bottom-line high vs low.

At 5.5 months, there were no significant associations between AL or nSES and high-fatigue, -pain, or - bother. Notably, high pain (6.08 [3.40–10.89]) and high fatigue (7.20 [4.27–12.15]) at baseline were strongly associated with a high burden of the respective symptom at 5.5 months; such an association was not observed with respect to baseline bother and bother at 5.5 months.

**Fig. 1:**
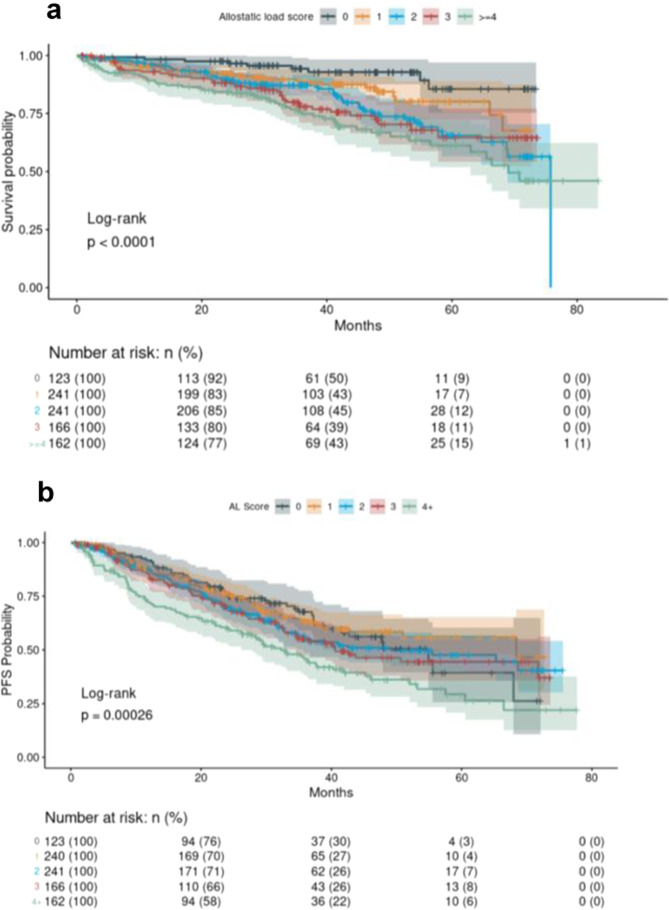
Examination of overall survival and progression-free survival by allostatic load score. **a** Overall survival by allostatic load score. **b** Progression-free survival by allostatic load.

**Fig. 2:**
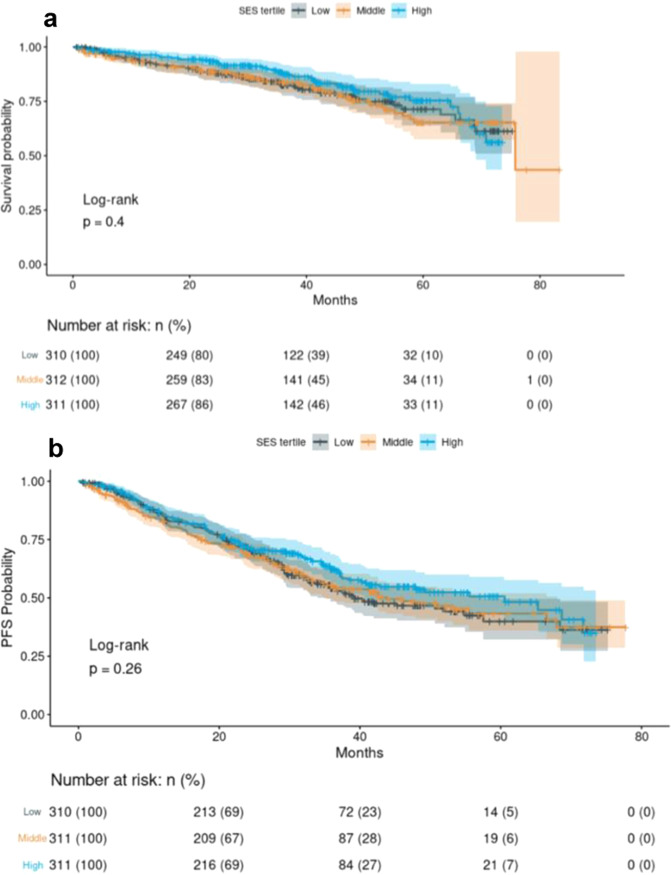
Evaluation of overall survival and progression-free survival by nSES. **a** Overall survival by nSES. **b** Progression-free survival by nSES.

There was borderline association between AL and non-completion of induction therapy (adjusted OR [95% CI] = 1.12 [1.00–1.25]), but no association between nSES and non-completion. Increasing AL was also associated with worse OS (log-rank *p* < 0.0001; Fig. 1) and PFS (log-rank *p* = 0.0003; Fig. 2). In adjusted Cox regression there was a 20% increase in the hazard of death from any cause (OS) for each one-unit increase in AL (hazard ratio [95% CI] = 1.20 [1.06–1.37]) (Table 3). However, a one-unit increase in AL was not significant to the hazard of a PFS event (1.08 [0.99–1.18]) (Table 4). There was no significant association between nSES and OS or PFS.

**Table 3:** Multivariable Cox regression for overall survival (*N* = 730).

	Hazard ratio (95% CI)	*P* value
Allostatic load	1.20 (1.06–1.37)	0.005
nSES	*Reference* = *Low*	0.39
Middle	1.25 (0.83–1.91)	
High	0.96 (0.62–1.51)	
Treatment Arm B (vs. *A*)	0.86 (0.61–1.21)	0.37
Age	1.03 (1.01–1.05)	0.01
Female Sex (vs. Male)	0.80 (0.56–1.14)	0.21
Race	*Reference* = *White*	0.10
Black	0.64 (0.30–1.36)	
Other	1.39 (0.50–3.91)	
Unknown	2.28 (1.10–4.74)	
ECOG Performance Status	*Reference* = *0*	0.07
1	1.46 (0.98–2.18)	
≥2	1.83 (1.04–3.24)	
Disease stage (ISS Stage)	*Reference* = *I*	0.02
II	1.95 (1.18–3.23)	
III	1.69 (0.97–2.95)	
Genetic high risk^a^	1.26 (0.89–1.81)	0.20
Baseline High Treatment Bother	0.93 (0.41–2.11)	0.86
Baseline High Pain	1.55 (1.06–2.28)	0.02
Baseline High Fatigue	1.28 (0.85–1.92)	0.24

^a^Genetic high risk is defined as the presence of either t(4;14) or -1q genetic abnormalities.

**Table 4:** Multivariable Cox regression for progression-free survival (*N* = 730).

	Hazard Ratio (95% CI)	*P* value
Allostatic load	1.08 (0.99–1.18)	0.09
nSES	*Reference* = *Low*	0.59
Middle	1.02 (0.77–1.35)	
High	0.89 (0.66–1. 19)	
Treatment Arm B (*vs. A*)	0.88 (0.70–1.11)	0.28
Age	1.01 (1.00–1.02)	0.21
Female Sex (*vs. Male*)	0.82 (0.65–1.04)	0.10
Race	*Reference* = *White*	0.35
Black	0.70 (0.45–1.09)	
Other	1.23 (0.54–2.79)	
Unknown	1.13 (0.60–2.14)	
ECOG Performance status	*Reference* = *0*	0.18
1	1.20 (0.93–1.54)	
≥2	1.43 (0.94–2.16)	
Disease stage (ISS Stage)	*Reference* = *I*	0.31
II	1.23 (0.92–1.64)	
III	1.25 (0.89–1.76)	
Genetic high risk^a^	1.16 (0. 91–1.47)	0.23
Baseline high treatment bother	0.91 (0.47–1.76)	0.77
Baseline high pain	1.29 (0.99–1.68)	0.06
Baseline high fatigue	1.19 (0.89–1.58)	0.24

^a^Genetic high risk is defined as the presence of either t(4;14) or -1q genetic abnormalities.

Results from the sensitivity analyses were largely similar to those from the primary analyses (not shown). Additionally, forward selection including the composite AL score and each of its individual constituents as predictors revealed that the composite score is superior to any one of the individual biomarkers when modeling OS and PFS, as it was selected into the model first. Bivariate associations between the study endpoints and each AL biomarker, individually, were explored in Supplementary Table 2.

## Discussion

In our study evaluating AL in patients with MM enrolled to E1A11, elevated AL was associated with high baseline fatigue and worse OS. Conversely, there was no significant relationship between AL and symptom burden at 5.5 months, non-completion of induction therapy or PFS. There was a relationship between nSES and baseline bother but there was no association between nSES and any remaining study endpoints. Taken together, these results suggest physiologic dysregulation secondary to chronic stressors, operationalized as AL, may have stronger implications for OS than some SDH at trial registration or diagnosis in patients with MM.

Our study is the first to examine the relationship between AL and OS or PFS in a cohort of only patients with MM. Study findings of an association between AL and overall survival are consistent with prior studies on AL in other cancer patients [17]. Specifically, previous studies suggest increasing AL is associated with worse overall and cancer-specific survival [16, 17]. Although, the relationship between AL and PFS did not reach statistical significance the direction of the relationship is consistent with the relationship of AL to survival in other oncologic studies [16, 17].

The clinical meaningfulness of AL needs to be contextualized within a patient’s values and goals for treatment. For example, a patient with a high AL may be interested in participating in psychosocial support services (e.g., stress reduction) that would reduce their AL and increase their overall survival. Specifically, the 20% increase in the risk of death from any cause with every 1 unit increase in AL, may be high enough threshold for them to consider AL risk-reducing strategies.

Currently, there are significant gaps in the literature on how or why elevated AL contributes to poorer outcomes among cancer patients. Possible explanations include a bifactor model wherein the individual biomarkers of AL affect outcomes independently, and through AL as a common factor [33]. The bifactor model may be a plausible pathway for how the biomarkers used in our AL measurement affect overall survival. Independently, C-reactive protein (CRP), albumin, creatinine, creatine clearance, BMI, and alkaline phosphatase have all been implicated in survival among patients with MM [34–38]. Elevated CRP, renal failure (elevated creatinine and/or creatinine clearance) and elevated alkaline phosphatase have been associated with worse mortality among patients with MM [34, 35, 37]. Moreover, low serum albumin is a poor prognostic indicator and is associated with a higher mortality in MM [35, 36, 39]. Some studies suggest underweight patients (BMI 18.5 kg/m^2^) have an increased mortality compared to healthy weight patients (18.5–24.9 kg/m^2^) [38]. Conversely, Kocoglu et al’s evaluation of the impact of BMI on survival after autologous stem cell transplantation indicates BMI does not significantly affect survival [40]. Although they noted those with morbid obesity trended toward a worse progression-free survival [40]. It is also possible that including a composite AL measure in these previous analyses may have mirrored our outcomes where a composite AL score has superior association to clinically relevant endpoints. Nevertheless, cumulatively, these results are consistent with our exploratory analyses suggesting associations between the majority of the biomarkers included in our AL composite measure and the study endpoint of overall survival.

AL is conceptualized as a composite measure of persistent activation of the HPA and the SAM secondary to external stressors or challenges such as financial hardship, childhood trauma, demanding workplace environments and low socioeconomic status [10, 41, 42]. Consequently, AL provides a novel avenue to understand the interaction between chronic adverse socially patterned exposures operationalized as negative SDH (e.g., poverty), their impact on physiology and implications on clinical outcomes. Currently, SDH data collection in clinical trials and in the non-clinical trial setting are usually a snapshot in time for example, at diagnosis or trial entry. However, our results suggest a snapshot approach may not be representative of the cumulative effects of adverse socially patterned exposures over a patient’s life span. For instance, Lee et al’s examination of lifetime SES and mortality among middle age and older age adults indicate that an upward trajectory from low SES to high SES, or a downward trajectory high SES to low SES, resulted in a worse mortality than starting and continuing at a high SES [43]. This suggests that upward social mobility may not mitigate some of the effects of earlier experiences living in deprivation (e.g., limited resources) [44]. Moreover, Krieger and colleagues showed residency in previously redlined neighborhoods (where biased loan lending and insurance practices based on neighborhood location or racial/ethnic composition were implemented [45]) is associated with advanced stages of breast, lung, cervical and colorectal cancer [46]. These studies suggest adverse socially patterned exposures have implications beyond the exposure timeframe and can persist throughout the life span; therefore, they need longitudinal evaluation through repeated measurements of potential biological corelates such as AL.

Study results of the relationship between baseline AL, pain, fatigue, and bother need to be interpreted with caution. The data source used for this study does not clarify if the reported symptoms are secondary to the patient’s disease burden or preexisting conditions. It should be noted that trial participants had a similar stage of disease, and the majority had an ECOG performance status of 0 or 1. Nevertheless, the relationship between baseline AL, symptom burden and induction non-completion are unclear and require further inquiry.

The finding of no association between nSES and overall survival is not consistent with prior studies in clinical trial populations that did not account for AL [47]. A possible explanation for this discrepancy includes differences in nSES indices between studies. It is feasible that nSES at trial registration does not capture the extent of socioeconomic hardship over a life span nor accurately reflect a change in nSES that could occur during treatment. Moreover, we may have captured the effect of nSES by analyzing AL as a separate domain. Consequently, these results further support more nuanced approaches to operationalize and conceptualize the effects of unfavorable SDH and their implications for clinical outcomes beyond the collection of zip code. The relationship between high or middle nSES and baseline bother are difficult to interpret within the context of no association between nSES and any symptom burden at 5.5 months.

An important limitation of this study was the availability of AL biomarkers in E1A11. Since E1A11 was not designed with AL as one of the study aims, the number of biomarkers for each of the physiological systems were limited. However, all the biomarkers used were reflective of the most common biomarkers currently used in the literature to measure AL [31]. Populations of patients who participate in clinical trials tend to be wealthier, younger and have high levels of educational achievement [48]. Consequently, the modest effects of AL on overall survival may not be generalizable to all populations; but rather represent the potential minimum effect with the actual effect potentially larger

## Conclusions

AL provides a much-needed framework to understand the cumulative effect of socially patterned exposures on clinical outcomes. To date, prior work has implicated elevated AL in unfavorable SDH, poor tumor prognostic features, and lower functional well-being scores on the Functional Assessment of Cancer Therapy-Breast Cancer survey [41, 49, 50]. Our study adds to this existing work and confirms that elevated AL at baseline has implications for clinical outcomes such as survival among multiple myeloma patients. Furthermore, we believe this study has laid the foundations for AL to be considered as a possible prognostic biomarker in conjunction with established prognostic markers and imaging, for overall survival in patients with MM.

## Supplementary information

Supplementary Tables

## Data Availability

The data from the present publication will be made available by request to the ECOG-ACRIN Cancer Research Group.
