# Supplementary material for: Examining allostatic load, neighborhood socioeconomic status, symptom burden and mortality in multiple myeloma patients
Source: Blood Cancer J. 2022 Apr 1;12(4):53. doi: 10.1038/s41408-022-00648-y (PMC8975964; doi:10.1038/s41408-022-00648-y)
Supplement: Supplementary file 1 — Supplementary Tables [file 41408_2022_648_MOESM1_ESM.docx]

**Supplementary Table 1: Biomarker cutoffs for dichotomization in Bivariate Analyses**

| **Biomarker** | **Cut-off** |
| --- | --- |
| BMI | underweight = <18.5,  overweight/obese ≥25 |
| Alkaline phosphatase | >147IU/L |
| Creatinine | >1.2mg/dL (Women)  >1.4mf/dL (Men) |
| Creatinine Clearance | <88mL/min (Women)  <97mL/min (Men) |
| C-reactive protein | ≥10mg/L |
| White Blood Cell Count | >1100 WBC/mL |
| Albumin | <3.4g/dL |

Supplementary Table 2: Bivariate Associations between the Study Endpoints and each AL Biomarker Individually

|  | **High Pain** | | **High Bother** | | **High Fatigue** | | **Induction**  **non-completion** | **Overall Survival** | **Progression-Free Survival** |
| --- | --- | --- | --- | --- | --- | --- | --- | --- | --- |
|  | **Baseline** | **5.5 months** | **Baseline** | **5.5 months** | **Baseline** | **5.5 months** |  |  |  |
| **†BMI** | - | - | - | - | 1.09  (0.99 - 1.19)  0.07 | - | - | 0.89  (0.79 - 1.00)  0.06 | - |
| **Alkaline Phosphatase** | - | - | - | - | 2.15  (1.16 - 4.01)  0.02 | - | - | 1.97  (1.16 - 3.34)  0.01 | - |
| **Creatinine** | - | - | - | - | 1.51  (1.05 - 2.17)  0.03 | - | - | 1.59  (1.14 - 2.22  0.01 | 1.25  (0.98 - 1.58)  0.07 |
| **Creatinine clearance** | - | - | - | - | - | - | - | 1.53  (1.10 – 2.15)  0.01 | - |
| **C-reactive protein** | 1.66  (0.94 - 2.97)  0.08 | - | - | - | - | - | - | 1.59  (0.97 - 2.63)  0.07 | - |
| **White blood cell count** | - | - | - | - | - | - | - | - | - |
| **Albumin** | 1.47  (1.07 – 2.03)  0.02 | - | - | - | 1.89  (1.35 - 2.66)  0.0002** | - |  | 1.85  (1.36 - 2.52)  <0.0001** | 1.37  (1.10 – 1.72)  0.006 |
| *All effects represent the comparison of patients with abnormal values for the given biomarker vs. patients with normal values, except BMI  **Statistically significant with Bonferroni correction ($\alpha$=0.05 with 63 tests)  †Per 5-unit increase  Cell structure: Top – odds/hazard ratio; Middle – 95% confidence interval; Bottom – P value | | | | | | | | | |

| Supplementary Table 3: Rates of High Symptom Burden at Baseline and 5.5 Months by AL | | | | |
| --- | --- | --- | --- | --- |
|  | Baseline | | 5.5 Months | |
|  | N (%, [95% CI*]) | N missing | N (%, [95% CI*]) | N missing |
| High Pain |  |  |  |  |
| AL=0 | 23 (19.0, [12.4 - 27.1]) | 2 | 8 (10.5, [4.7 - 19.7]) | 47 |
| AL=1 | 83 (35.0, [29.0 - 41.5]) | 4 | 24 (17.0, [11.2 - 24.3]) | 100 |
| AL=2 | 86 (37.1, [30.8 - 43.6]) | 9 | 25 (18.4, [12.3 - 25.9]) | 105 |
| AL=3 | 61 (37.7, [30.2 - 45.6]) | 4 | 13 (14.4, [7.9 - 23.4]) | 76 |
| AL>=4 | 67 (43.5, [35.5 - 51.7]) | 8 | 15 (16.7, [9.6 - 26.0]) | 72 |
| High Bother |  |  |  |  |
| AL=0 | 0 (0.0, [0.0 - 3.2]) | 9 | 9 (11.7, [5.5 - 21.0]) | 46 |
| AL=1 | 9 (3.9, [1.8 - 7.3]) | 11 | 19 (13.4, [8.3 - 20.1]) | 99 |
| AL=2 | 10 (4.6, [2.2 - 8.3]) | 24 | 17 (12.5, [7.5 - 19.3]) | 105 |
| AL=3 | 4 (2.6, [0.7 - 6.6]) | 15 | 9 (9.9, [4.6 - 17.9]) | 75 |
| AL>=4 | 7 (4.8, [1.9 - 9.6]) | 15 | 9 (10.0, [4.7 - 18.1]) | 72 |
| High Fatigue |  |  |  |  |
| AL=0 | 16 (13.2, [7.8 - 20.6]) | 2 | 16 (20.8, [12.4 - 31.5]) | 46 |
| AL=1 | 55 (23.4, [18.1 - 29.3]) | 6 | 36 (25.2, [18.3 - 33.1]) | 98 |
| AL=2 | 62 (26.7, [21.1 - 32.9]) | 9 | 28 (20.6, [14.1 - 28.4]) | 105 |
| AL=3 | 39 (24.1, [17.7 - 31.4]) | 4 | 23 (25.0, [16.6 - 35.1]) | 74 |
| AL>=4 | 55 (35.9, [28.4 - 44.1]) | 9 | 16 (17.8, [10.5 - 27.3]) | 72 |
| *Exact binomial confidence interval | | | | |

| Supplementary Table 4: Rates of High Symptom Burden at Baseline and 5.5 Months by nSES | | | | |
| --- | --- | --- | --- | --- |
|  | Baseline | | 5.5 Months | |
|  | N (%, [95% CI*]) | N missing | N (%, [95% CI*]) | N missing |
| High Pain |  |  |  |  |
| Low | 116 (38.4, [32.9 - 44.2]) | 8 | 31 (16.4, [11.4 - 22.5]) | 121 |
| Middle | 116 (37.9, [32.4 - 43.6]) | 6 | 31 (17.9, [12.5 - 24.5]) | 139 |
| High | 88 (29.5, [24.4 - 35.1]) | 13 | 23 (13.5, [8.7 - 19.5]) | 140 |
| High Bother |  |  |  |  |
| Low | 5 (1.7, [0.6 - 4.0]) | 22 | 23 (12.1, [7.8 - 17.6]) | 120 |
| Middle | 12 (4.2, [2.2 - 7.1]) | 23 | 21 (12.1, [7.6 - 17.9]) | 138 |
| High | 13 (4.6, [2.5 - 7.8]) | 29 | 19 (11.0, [6.8 - 16.7]) | 139 |
| High Fatigue |  |  |  |  |
| Low | 73 (24.3, [19.5 - 29.5]) | 9 | 42 (22.1, [16.4 - 28.7]) | 120 |
| Middle | 84 (27.7, [22.8 - 33.1]) | 9 | 42 (24.3, [18.1 - 31.4]) | 139 |
| High | 70 (23.4, [18.7 - 28.6]) | 12 | 35 (20.0, [14.3 - 26.7]) | 136 |
| *Exact binomial confidence interval | | | | |
